# MT1G is Silenced by DNA Methylation and Contributes to the Pathogenesis of Hepatocellular Carcinoma: Erratum

**DOI:** 10.7150/jca.104262

**Published:** 2024-10-25

**Authors:** Ju-deng Zeng, Ning Zhang, Gui-jun Zhao, Li-xia Xu, Yang Yang, Xiao-yi Xu, Meng-ke Chen, Hui-yun Wang, Xiao-xing Li

**Affiliations:** 1 State Key Laboratory of Oncology in South China, Collaborative Innovation Center for Cancer Medicine, Sun Yat-Sen University Cancer Center, Guangzhou, Guangdong, China; 2 Department of Gastroenterology, The First Affiliated Hospital, Sun Yat-sen University, Guangzhou, Guangdong, China; 3 Department of Gastroenterology and Hepatology, Inner Mongolia People's Hospital, Hohhot, Inner Mongolia Autonomous Region, China

In our published paper, we regret to identify that the images shown in Figures 3B-D, 4B-D, 5D, and 5H were inadvertently misused during the selection of representative images from a large amount of data. The corrected versions of Figures 3B-D, 4B-D, 5D, and 5H are provided below. The authors confirm that the corrections made in this erratum do not alter the overall conclusions of the study, and we sincerely apologize for any mistakes and any inconvenience caused.

## Figures and Tables

**Fig 3:**
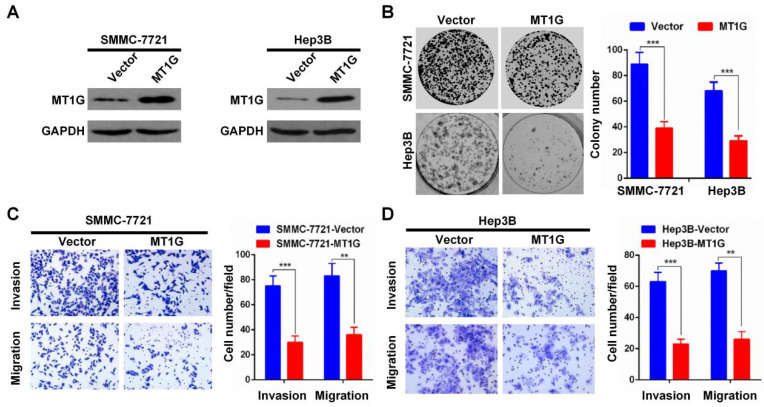
Corrected image as shown

**Fig 4:**
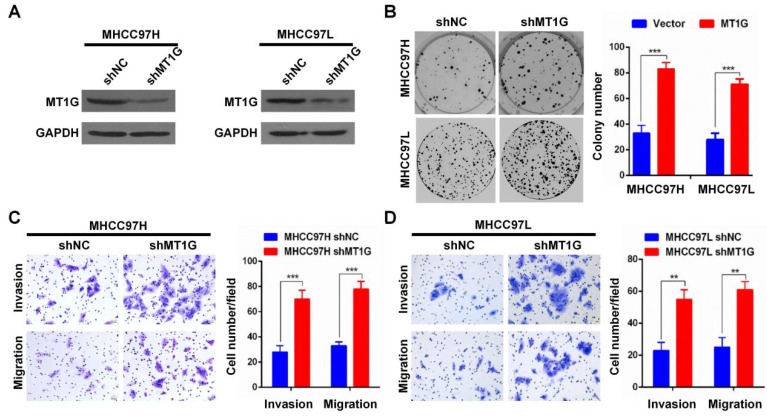
Corrected image as shown

**Fig 5:**
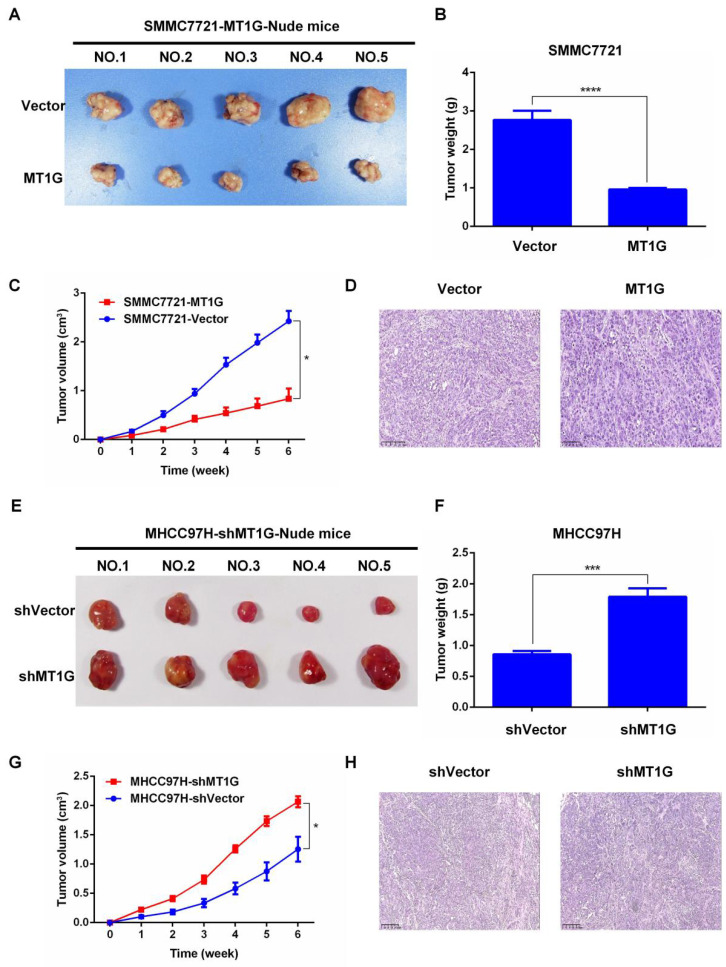
Corrected image as shown

